# High-Quality Genomes of Pangolins: Insights into the Molecular Basis of Scale Formation and Adaption to Myrmecophagous Diet

**DOI:** 10.1093/molbev/msac262

**Published:** 2022-12-31

**Authors:** Dingyu Yan, Xier Luo, Jiabin Tang, Shanghua Xu, Kongwei Huang, Xiaobo Wang, Tong Feng, Tengcheng Que, Miaomiao Jia, Xiaobing Guo, Saif ur Rehman, Zhipeng Li, Yufeng Yang, Kaixiang Li, Kuiqing Cui, Jue Ruan, Qingyou Liu

**Affiliations:** Guangxi Forestry Research Institute, 530002 Nanning, China; Guangdong Provincial Key Laboratory of Animal Molecular Design and Precise Breeding, School of Life Science and Engineering, Foshan University, 528225 Foshan, China; Shenzhen Branch, Guangdong Laboratory of Lingnan Modern Agriculture, Genome Analysis Laboratory of the Ministry of Agriculture and Rural Affairs, Agricultural Genomics Institute at Shenzhen, Chinese Academy of Agricultural Sciences, 518120 Shenzhen, China; State Key Laboratory for Conservation and Utilization of Subtropical Agro-Bioresources, Guangxi University, 530005 Nanning, China; Guangxi Forestry Research Institute, 530002 Nanning, China; Guangdong Provincial Key Laboratory of Animal Molecular Design and Precise Breeding, School of Life Science and Engineering, Foshan University, 528225 Foshan, China; State Key Laboratory for Conservation and Utilization of Subtropical Agro-Bioresources, Guangxi University, 530005 Nanning, China; Shenzhen Branch, Guangdong Laboratory of Lingnan Modern Agriculture, Genome Analysis Laboratory of the Ministry of Agriculture and Rural Affairs, Agricultural Genomics Institute at Shenzhen, Chinese Academy of Agricultural Sciences, 518120 Shenzhen, China; State Key Laboratory for Conservation and Utilization of Subtropical Agro-Bioresources, Guangxi University, 530005 Nanning, China; Shenzhen Branch, Guangdong Laboratory of Lingnan Modern Agriculture, Genome Analysis Laboratory of the Ministry of Agriculture and Rural Affairs, Agricultural Genomics Institute at Shenzhen, Chinese Academy of Agricultural Sciences, 518120 Shenzhen, China; State Key Laboratory for Conservation and Utilization of Subtropical Agro-Bioresources, Guangxi University, 530005 Nanning, China; Guangxi Terrestrial Wildlife Rescue Research and Epidemic Disease Monitoring Centre, 530003 Nanning, China; Guangxi Forestry Research Institute, 530002 Nanning, China; Guangxi Forestry Research Institute, 530002 Nanning, China; State Key Laboratory for Conservation and Utilization of Subtropical Agro-Bioresources, Guangxi University, 530005 Nanning, China; State Key Laboratory for Conservation and Utilization of Subtropical Agro-Bioresources, Guangxi University, 530005 Nanning, China; State Key Laboratory for Conservation and Utilization of Subtropical Agro-Bioresources, Guangxi University, 530005 Nanning, China; Guangxi Forestry Research Institute, 530002 Nanning, China; Guangdong Provincial Key Laboratory of Animal Molecular Design and Precise Breeding, School of Life Science and Engineering, Foshan University, 528225 Foshan, China; Shenzhen Branch, Guangdong Laboratory of Lingnan Modern Agriculture, Genome Analysis Laboratory of the Ministry of Agriculture and Rural Affairs, Agricultural Genomics Institute at Shenzhen, Chinese Academy of Agricultural Sciences, 518120 Shenzhen, China; Guangdong Provincial Key Laboratory of Animal Molecular Design and Precise Breeding, School of Life Science and Engineering, Foshan University, 528225 Foshan, China

**Keywords:** pangolin, genome assembly, scale formation, myrmecophagous diet, evolution

## Abstract

Pangolins are one of nature's most fascinating species being scales covered and myrmecophagous diet, yet relatively little is known about the molecular basis. Here, we combine the multi-omics, evolution, and fundamental proteins feature analysis of both Chinese and Malayan pangolins, highlighting the molecular mechanism of both myrmecophagous diet and scale formation, representing a fascinating evolutionary strategy to occupy the unique ecological niches. In contrast to conserved organization of epidermal differentiation complex, pangolin has undergone large scale variation and gene loss events causing expression pattern and function conversion that contribute to cornified epithelium structures on stomach to adapt myrmecophagous diet. Our assemblies also enable us to discover large copies number of high glycine-tyrosine keratin–associated proteins (HGT-KRTAPs). In addition, highly homogenized tandem array, amino content, and the specific expression pattern further validate the strong connection between the molecular mechanism of scale hardness and HGT-KRTAPs.

## Introduction

Pangolins are one of nature's most fascinating species being uniquely constructed (Challender et al. 2019). They pertain to a monophyletic clade exclusive of other living placentals comprise the order Pholidota (Szalay 2005). Molecular phylogenetic analysis indicate that their closest relatives are the carnivores, yet the unique morphology, including their epidermal scales and anatomical adaptations to myrmecophagous diet, has been characterized highly distinctive to other mammals (Mitra 1998; Kingdon and Hoffmann 2013).

Pangolins are the few living mammals that have developed nail-like structures on their body to defend itself from sharp threats even for tigers and lions (Challender et al. 2019). Scales are keratinized extrusions of the epidermis, consisting of flat, solid, and keratinized cells, and enriched in glycine (∼11.2%) and tyrosine (∼18.6%) residues (Spearman 1967; Gao et al. 1989; Tong et al. 1995). X-ray diffraction (XRD) analysis on pangolin scales exhibited both α- and β-keratin in which β-keratin is only observed in the skin appendages of Sauropsida (Wilson 1994). Recent molecular evolutionary analysis determined the sequences of most of keratins (KRTs) and keratin-associated proteins (KRTAPs) and identified pangolin-specific amino acid variations in functionally relevant domains on *KRT36*, *KRT75*, *KRT82*, and *KRTAP3-1* suggesting potential contribution to the development of scales (Meyer et al. 2013; Choo et al. 2016). Proteomics and transcriptomics analysis on scale and hair exhibited a more complex signaling pathways related to immune and keratinocyte differentiation and further demonstrated elevated abundance of *KRT36* in scale (Li et al. 2020). However, only enrichment of KRT proteins could not explain the extremely high component of glycine and tyrosine in pangolin scale indicating unconfirmed essential components (Wang and Sullivan 2017).

Another unusual feature of pangolins is the myrmecophagous and termitophagous diet. The digestive tract of the pangolin contains a number of anatomical and microbial composition characteristics, such as thick cornified stratified squamous epithelium lining on the stomach, loss on teeth, and host-associated microbiomes. Acidic mammal chitinase has been identified in the pangolin stomach closely related to the digestive function of pangolins (Ma et al. 2017). Recently, convergent evolution of myrmecophagous mammals, including anteaters, echidnas and pangolins, has been identified for the interactions between hosts and the gut microbiota to increase trehalose utilization and toxin resistance (Cheng et al. 2022). The presence of thick cornified epithelium on stomach is another important feature for pangolins to facilitate the grinding process by swallowing small stones and soil during feeding. Molecular researches relating to pangolin stomach cornification has not been conducted yet. We stress on a conserved loci among mammals, epidermal differentiation complex (EDC), containing most important structural proteins in the cornified epithelium (Alibardi 2016). Pangolin stomach cornification could be well researched by evaluating whether conserved tissues specification reversion occurred on the EDC and related genes.

Fragmental genome sequences and incomplete genome annotation are the primary hinder to deeper dig in the complex regions such as the EDC and KRTAP clusters. Here, we generate two high-quality genomes for Chinese and Malayan pangolin and largely improve the quality of protein annotation especially those with enriched expression manner on skins. We use the transcriptome data to generate comprehensive pangolin tissues expression pattern for all 22 tissues in order to identify various tissues specialized genes. We then compare the EDC organization and the tissues specification of EDC genes among pangolin and other mammals, suggesting DNA variations derived expression pattern change on stomach samples. Most strikingly, we identify a huge number of pangolin-specific and highly specialized high glycine-tyrosine keratin–associated proteins (HGT-KRTAPs), severely ignored in previous researches, contributing to the hardness character of pangolin scale validated by the expression pattern and amino acid contents. In summary, it is the first time to emphasize the role of EDC and HGT-KRTAPs in the myrmecophagous diet and scale hardness in pangolin researches, which could be served as molecular evolutionary insights into the novel phenotypic formation.

## Results

### Nanopore Long Reads–Based Pangolin High Quality Assemblies

**Fig. 1.**
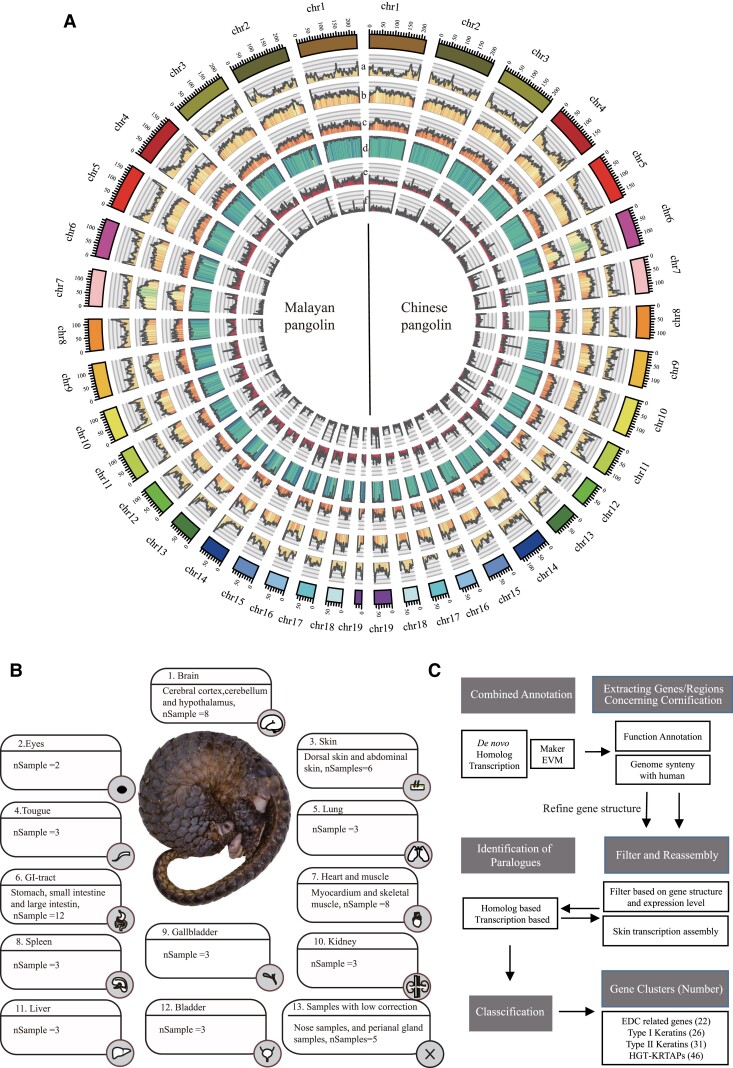
Landscape of the Chinese and Malayan pangolin genomes. (*A*) Integration of genomic data using 2 Mb bins in Hi-C assembled pseudo-chromosomes. (*a*) Distribution of the GC content (GC content >34% and <58%); (*b*) distribution of the repeat sequences percentage >0% and <80%; (*c*) distribution of the long interspersed element L1 (LINE-L1) percentage >0% and <80%; (*d*) percent of the genome with similarity above 90% between our assemblies to previous versions; (*e*) distribution of the heterozygosity density of our sample (percentage >0% and <1%); (*f*) distribution of the assembly error sites density of our samples (percentage >0% and <0.01%). (*B*) The tissues analyzed in this study, complemented with RNA-sequencing data for 19 different Malayan pangolin tissues with total 62 samples. (*C*) Pipeline of manual annotation for KRTs, HGT-KRTAPs, and EDC genes. We firstly screen the annotated genes concerning cornification according to the function annotation and the collinearity with known regions. We then filter unqualified annotated gene according to exon–intron organization and reannotated based on the transcription assembly from skin samples. The newly annotated gene amino acid sequences are further selected as input to iteratively identify paralog genes.

The blood samples from a female Malayan pangolin (*Manis javanica*, MJ) and a female Chinese pangolin (*Manis pentadactyla*, MP) are used for sequencing (supplementary table S1, Supplementary Material online). Multiple sequencing technologies are applied to guarantee the integration and accuracy: 1) Oxford Nanopore long reads (∼108× depth for MJ and ∼104× depth for MP) with PromethION platform and 2) paired-end reads with Illumina platform (supplementary table S2, Supplementary Material online). The contig assembly is performed by Wtdbg2 (Ruan and Li 2020) using Nanopore long reads and then polished by Nextpolish using both long and short sequencing reads, resulting to a total length of 2.44 and 2.54 Gb, slightly larger than estimated genome size using Illumina reads and contig N50 sizes of 15.81 and 13.97 Mb for Malayan and Chinese pangolins, respectively (Table 1, supplementary fig. S1, Supplementary Material online, supplementary tables S3 and S4, Supplementary Material online). To acquire scaffolds level assembly, we use chromosome conformation capture sequencing (Hi-C) data to construct pangolin pseudo-chromosomes covering more than 96% of the genomes (fig. 1*A*, supplementary figs. S2 and S3, Supplementary Material online, supplementary table S5, Supplementary Material online).

**Table 1.** Summary Statistics for the Genome Sequences.

	Chinese pangolin	Malayan pangolin
Genome		
Total genome size (Mb)	2,537	2,443
Contig number	6,002	2,568
Contigs N50 (Mb)	14.0	15.8
Contigs L50	50	47
Longest contig (Mb)	53.2	55.9
Pseudo-chromosomes Number	18	18
Percentage of Pseudo-chromosomes	97.0%	98.4%
Quality		
Error rate^a^	1.76 × 10^−5^	9.15 × 10^−6^
Coverage (≥10×)	98.9%	99.2%
Repeat content	44.2%	42.2%
BUSCO assessment^b^	95.7%	96.2%

a Error rate is calculated by the homozygous variants divided by length of the whole genome where homozygous variants are generated by mapping paired-end reads to our assembly.

b BUSCO assessment is performed with transcriptome mode in the software.

The nucleotide accuracy is high in our assembly, in which the error rates of Malayan and Chinese pangolin genomes were 9.15 × 10^−6^ and 1.76 × 10^−5^. QUAST analysis (Mikheenko et al. 2018) also indicates a high mapping and coverage rate using both short and long sequencing reads in both genomes, in which more than 98.9% of sequences with more than 10× depth in both genomes (supplementary table S6, Supplementary Material online). The previous de novo pangolin assemblies (YNU_ManJav_2.0 and YNU_ManPten_2.0) were firstly assembled from Illumina HiSeq reads and then scaffolded using 10× genomic reads. Compared with previous assemblies (scaffold version), our assemblies (contig version) have larger genome sizes, comparative N50 length, higher resolution of repetitive sequences, and much fewer gaps. Pangolin genomes contain long repeats that are difficult to span by using short sequence reads, resulting breaks in sequence contiguity. Our assemblies identify significant higher rate of repeat sequences than previous version (42.2% vs. 33.5% and 44.2% vs. 37.1% in Malayan and Chinese pangolin) (supplementary table S7, Supplementary Material online). The most abundant newly identified repeat elements are long interspersed nuclear element LINE/L1, which account for 78–82% of total increment between different version of no-gapped genome sequences. Additionally, our assemblies have higher reliability on the accuracy compared with scaffolded sequences. For example, the current assemblies have the longest nongapped contigs over 55 Mb, over 50 times longer than previous version.

### Improved Gene Annotation

To acquire high quality of protein annotation, we additionally sequence ∼3.3 billion RNA-seq reads from 22 different pangolin tissues with 2–3 replications (supplementary table S8, Supplementary Material online, supplementary fig. S4, Supplementary Material online). Combing de novo/homolog/RNA-seq prediction, a total of 21,363/20,449 protein-coding genes were annotated in the Malayan and Chinese pangolin genomes (contig level) (fig. 1*B*, supplementary fig. S5, Supplementary Material online, supplementary table S9, Supplementary Material online). BUSCO assessment (Waterhouse et al. 2018) indicated that the genomes are 96.2% and 95.7% complete in 4,104 single copy mammalian orthologous genes, underscoring the improvement of the gene-structure predictions compared with previous assemblies (supplementary table S10, Supplementary Material online).

To further identify genes concerning the mechanism of cornification and scale formation, we reannotate genes in four distinct regions: the EDC, type I/II keratin gene clusters, and HGT-KRTAPs cluster, in Malayan pangolin genome (fig. 1*C*). We firstly screen the annotated genes above to determine candidate contigs and regions of these genes. According to types of exon–intron organization in human annotation, we filter unqualified initial annotated gene and reannotated based on the transcription assembly from abdominal and dorsal skin samples or homology-based gene model by genBlastG (She et al. 2011). The newly annotated gene amino acid sequences are further selected as input to iteratively identify paralogue genes. The final gene set and function annotation were edited by manually. In total, we identified 22 genes homolog to human EDC region, 26 genes in type I keratin gene cluster, 31 genes in type II keratin gene cluster, and 45 HGT-KRTAPs in Malayan pangolin genome.

### Cornification in Pangolin Stomach

**Fig. 2.**
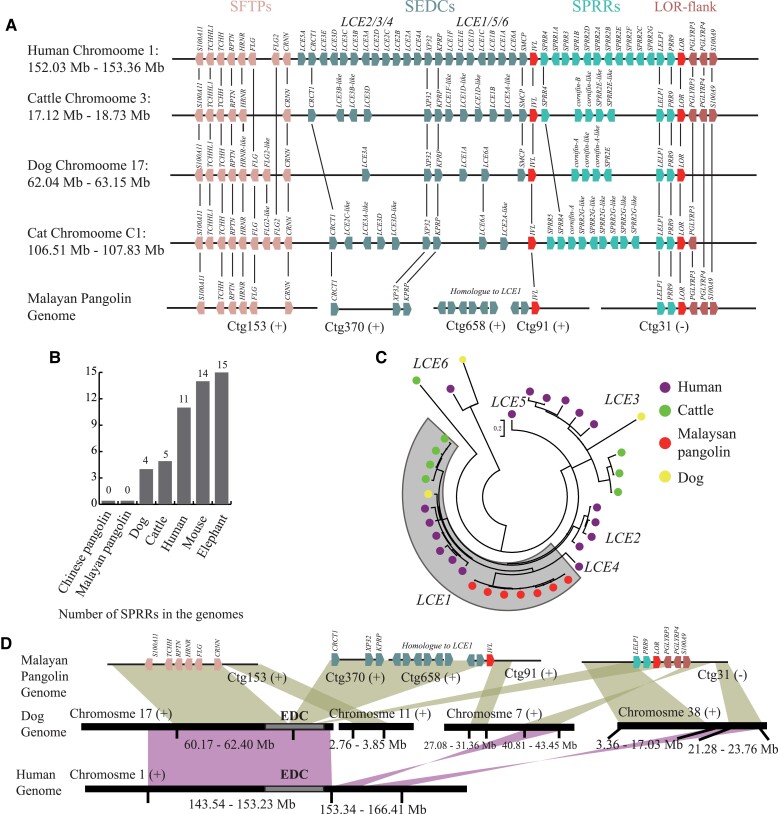
Organization of the EDC gene cluster in pangolin genome. (*A*) EDC structures among human, cattle, dog, cat, and Malayan pangolin (ctg153, ctg370, ctg658, ctg91, and ctg31) are schematically depicted. Homologs are lined between them. Arrows indicate the orientation of the genes. Note that the schemes are not drawn to scale. (*B*) The number of SPRRs among the genomes of pangolins and other species. (*C*) Phylogenetic tree of LCEs in Malayan pangolin, human, dog, and cattle. (*D*) Genome synteny among human, dog, and Malayan pangolin. Green blocks indicate credible synteny regions between pangolin and dog, and red blocks indicate credible synteny regions between human and dog.

Cornification is an important step of stratum corneum formation at the conversion of living epithelial cells to dead corneocytes, which contributes to pangolin stomach affording the mucosa protection from mechanical abrasion during mastication, and consists of three major steps: formation of the intracellular keratin network, cornified envelopes, and intercellular lipids (Nisa et al. 2010; Matsui and Amagai 2015). In mammals, EDC genes specifically express in the cornification process playing an important role in the first two steps of cornification. They can be classified as loricin, involucrin, small proline-rich (SPRR) proteins, late cornified envelope (LCE) proteins, and the S100-fused type proteins (SFTPs) (Kypriotou et al. 2012). In the genome of Malayan pangolin, the organization of EDC has multiple variations on the gene order and gene number compared with human, cattle, dog, and cat (fig. 2*A*). The pangolin EDC can be divided to three functional regions: 1) SFTP gene cluster identified in the ctg153, including *CRNN*, *FLG*, *HRNR*, *RPTN*, and *TCHH*; 2) LOR-flank gene cluster, including *S100A9*, *PGLYRP4*, *PGLYRP3*, *LOR*, *PPR9*, and *LELP*, in the ctg31; and 3) simple EDC (SEDC) gene cluster, including *IVL* in ctg91, *LCE* homolog genes in ctg658, and *KPRP*, *LEP7*, and *CRCT1* in ctg370. One interesting gene loss event is the decay of SPRR proteins (fig. 2*B*). SPRRs are important to constitute cornified cell envelope precursors, and the expression of *SPRR4* and *SPRR2G* is intensely induced during differentiation and after UV treatment, which are supposed to provide protection against UV-induced DNA damage (Henry et al. 2012). Pangolins have overlapping scales covering the body and mainly shelter in burrows, resulting in limited exposure of sunlight. The coelimination could be due to relaxed constraint mediating pseudogenization or genome variation mediating gene loss for their location around breakpoints (Albalat and Cañestro 2016). Besides, the number of Malayan pangolin LCEs are reduced to 8, compared with 18 in human. LCE genes can be divided into three main groups designated *LCE1*, *LCE2*, and *LCE3* as well as three individual LCE genes (*LCE4A*–*LCE6A*). Phylogenetic analysis indicates that all Malayan pangolin *LCEs* are homolog to *LCE1* (fig. 2*C*). Although pangolin ctg370 is syntenic with the DNA sequences of *LCE2/3/4* cluster in other mammals, we do not find any intact gene model supported by the annotation based on transcriptome and homolog protein. Relative mammals have at least one copy of *LCE2/3/4*, indicating a more radical pseudogenization in pangolin ancestor. We further identified pangolin-specific variation in EDC contrasting with continuous and integrate conserved structure of EDC in other mammals (fig. 2*D*). Malayan pangolins have accordant synteny relationship in the both sides of EDC, including the upstream of SFTPs and the downstream of LOR-flank. However, the downstream of SFTPs in Ctg153 (+) and SEDC in Ctg91 (+) have a high synteny relationship with dog chromosome 11 and chromosome 7, respectively, and the upstream LOR-flank in Ctg31 (−) have a high synteny relationship with dog chromosome 38. The complex genome relationship indicates that the inside of Malayan pangolin EDC has been divided into at least three nonsynteny regions exactly corresponding to their functions.

**Fig. 3.**
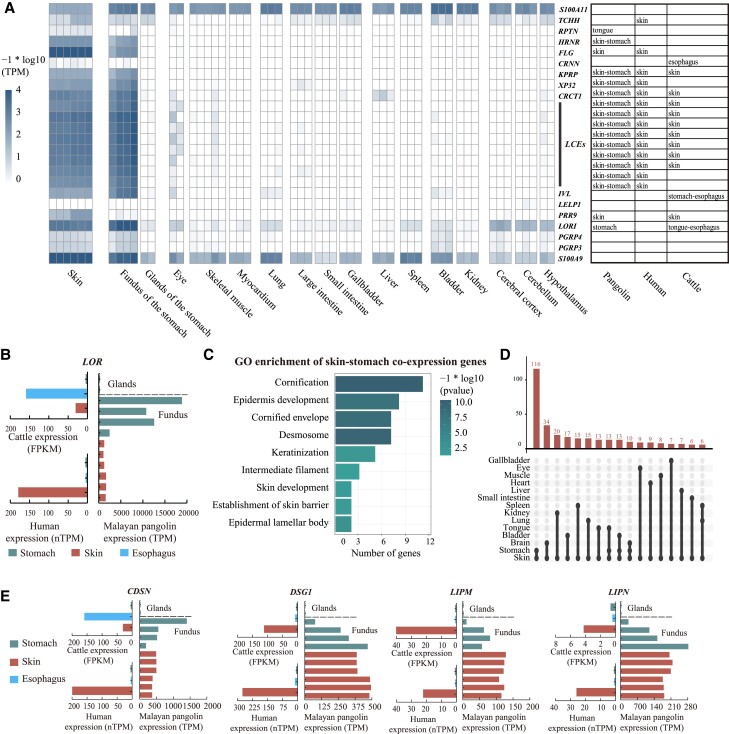
Tissues specification of EDC genes in pangolin. (*A*) Expression (TPM, log10-transformed) of genes across tissues. The tissues are shown in bottom, and genes are shown in right. The chart represents the tissue specification of human and pangolin respectively. (*B*) Expression of LOR in the stomach, skin, and esophagus. Vertical line indicates the samples and horizontal line indicates expression level. The data of human and cattle are derived from human protein atlas and Ruminant Genome Database. The stomach expression of cattle is the highest value among rumen, reticulum, omasum, and abomasum. The fundus (*n* = 4) and gland (*n* = 2) in pangolin stomach represent two location of the organ, where fundus has thick cornified stratified squamous epithelium yet gland lacking. TPM, transcript per million; nTPM, normalized TPM within each data source using Trimmed mean of *M* values, FPKM: fragments per kilobase of transcript per million reads mapped. (*C*) GO enrichment of pangolin skin-stomach co-expression genes. (*D*) The distribution of pangolin co-expression gene number related to skin or stomach. The tissues with co-expression pattern are labeled with dots in the bottom and connected by lines. The gene numbers are shown in the histogram. (*E*) Expression (TPM, nTPM, or FPKM) of CDSN, DSG1, LIPM, and LIPN in the stomach, skin, and esophagus. The layout is same with 2*B*.

Changes on genes expression are another obvious influence among the Malayan pangolin EDC. Benefitted from the transcriptome of Malayan pangolin major tissues or organs, we perform a tissues specification analysis based on the distribution of mRNA levels and divide them into 1) tissue-enriched genes with mRNA levels in one tissue type at least five times the maximum levels of all other analyzed tissues and 2) coexpression (group-enriched) genes with enriched expression in a small number of tissues, similar with previous human classification criteria (supplementary fig. S6, Supplementary Material online, supplementary tables S11 and S12, Supplementary Material online) (Uhlén et al. 2015). By comparing the tissues specification of EDC genes between human and pangolin, we find that pangolin have more skin-stomach co-expression or stomach-enriched genes that are skin enriched in human, resulting an excess expression on stomach especially for those samples at fundus (fig. 3*A*). For example, loricrin (*LORI*) is identified skin-enriched in human with highest expression in human skin and in cattle esophagus following undetected expression on various stomach tissues on both species (Uhlén et al. 2015; Chen et al. 2019), while *LORI* has highest expression level in pangolin stomach classified as stomach-enriched (fig. 3*B*). In general, more than half of genes in EDC with higher expression on stomach than skin, including genes encoding components of the cornified cell envelope (*IVL*, *LORI*, *LCEs*) (Henry et al. 2012). Genome variation on conserved noncoding elements (CNEs) could influence gene expression. We collect previous identified 48 EDC CNEs (de Guzman Strong et al. 2010) and align them to the genome of Malayan pangolin, dog, and cattle. Based on the mapping quality of CNEs, we found more CNEs are likely decayed in Malayan pangolin in which four CNEs (ID: 639, 877, 964, and 1040) have <50% coverage in pangolin and more than 90% in other species corresponding to one CNE (ID: 555) in dog and zero in cattle with the same cutoff (supplementary table S13, Supplementary Material online). Moreover, there are 60% of CNEs in SEDC cluster are critically decayed, contrasting to 20% and 30% in dog and cattle respectively. The locations of decayed CNEs also accord with the genes with expression reversion in stomach that inclined to locate at the SEDCs and boundary of LOR-flank, further supporting variations in the network of interspersed regulatory elements to gene expression.

Cornification is an important step of stratum corneum formation at the conversion of living epithelial cells to dead corneocytes and consists of three major steps: formation of the intracellular keratin network, cornified envelopes, and intercellular lipids (Matsui and Amagai 2015). The skin-stomach coexpression genes further validate that pangolin has an additional cornification formation pathway on stomach cells affording the mucosa protection from mechanical abrasion during mastication. We identify 391 skin-related group-enriched in Malayan pangolin in which skin-stomach coexpression genes have the largest number (*n* = 104). Gene ontology (GO) analysis indicate that these genes are significantly related to the barrier protection, including “cornification” (*P* = 3.0E − 11), “cornified envelope” (*P* = 1.5 − 09), etc. (fig. 3*C*and*D*, supplementary table S14, Supplementary Material online). Except EDC genes, we identify seven genes with both high level expression in Malayan pangolin stomach and skin, yet skin-enriched in human, participating the second and third step of cornification (*CDSN*, *LIPM*, *LIPN*, *DSG1*, *KLK5*, *DMKN*, and *DNASE1L2*) (fig. 3*E*, supplementary fig. S7, Supplementary Material online). For example, *CDSN* (corneodesmosin) and *DSG1* (desmoglein 1) localized in the intracellular space of the stratum corneum as components of the corneodesmosome to help adhesion functions (Matsumoto et al. 2008; Samuelov et al. 2013). *LIPM* and *LIPN* are supposed to participate in the establishment of the barrier function by catalyzing the maturation of extracellular lipids (Toulza et al. 2007).

Although the expression data are composed of multiple cell types of Malayan pangolin stomach, the specific expression still provide a strong molecular evidence on function specification to adapt absence of dentition and their diet exclusively on termites and ants.

### Comparative Analyses Identifying Pangolin-Specific HGT-KRTAP Gene Expansion

Previous study indicated that there are 18 amino acids in pangolin scales, enriched in glycine (∼11.2%) and tyrosine (∼18.6%) (supplementary fig. S8, Supplementary Material online) (Tong et al. 1995). In fact, pangolin scale may be the tissue with most abundant tyrosine, versus to 2.0% in hair, 6.4% in claw, and 4.7–10.4% in quill of other species (Gillespie 1990). To dissect the genetic underpinnings of scale formation in pangolin, we identify 64 proteins with high proportion of tyrosine residues (the number of tyrosine residues divided by all number of residues in per proteins, cutoff = 5%) and skin-enriched expression pattern in Malayan pangolin, in which 48 proteins are HGT-KRTAPs indicating that HGT-KRTAPs could be the most important functional and constituent protein in pangolin scale.

**Fig. 4.**
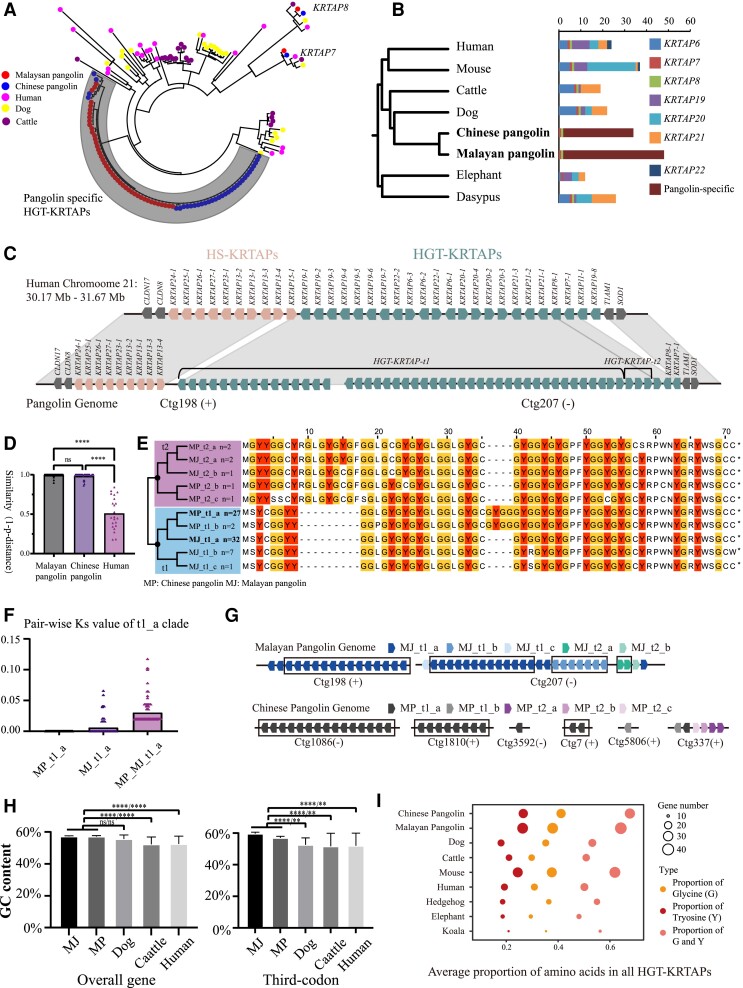
Organization and character of the HGT-KRTAPs gene cluster in pangolin genome. (*A*) Phylogenetic tree of all HGT-KRTAPs among human, dog, cattle, and two pangolins. Box indicates pangolin specific HGT-KRTAPs. (*B*) Mammal species tree constructed by genome-wide single copy genes and the number of HGT-KRTAPs subfamilies in each species. (*C*) Genome synteny between human (chromosome 21) and Malayan pangolin (ctg198 and ctg207) are schematically depicted. Arrows indicate the orientation of the genes. Note that the schemes are not drawn to scale. (*D*) Similarity distribution of neighboring HGT-KRTAPs within human and pangolins. The height of histogram indicates the mean similarity. Similarity is calculated by 1-*p*-distance between aligned proteins. Height of column indicates the mean value of similarity. The similarity values of each neighboring proteins are plotted with dots. Significant differences based on one-way ANOVA are indicated with **** (*P* < 0.0001). (*E*) Evolutionary relationships among nonduplicated HGT-KRTAPs. MP, Chinese pangolin; MJ, Malayan pangolin; t1, t2, types of pangolin-specific HGT-KRTAPs; a, b, c: protein clades in each types. (*F*) Ks distribution within and between t1_a clades of both pangolins. The height of histogram indicates the mean Ks. MP_t1_a, Ks distribution of t1_a within Chinese pangolin; MJ_t1_a, Ks distribution of t1_a clade within Malayan pangolin; MP_MJ_t1_a, Ks distribution of t1_a clades between of Chinese pangolin and Malayan pangolin. (*G*) The organization of HGT-KRTAPs. Neighboring genes with identical CDS are plot in box. (*H*) The GC content of HGT-KRTAPs among pangolins, dog, cattle, and human. Significant differences based on one-way ANOVA are indicated with **** (*P* < 0.0001), ** (*P* < 0.01), or ns (*P* > 0.05). (*I*) Average proportion of glycine, tyrosine, and glycine-tyrosine residues among all HGT-KRTAPs in species.

The gene families analysis among pangolins and relative species further indicate that HGT-KRTAPs of both pangolins have undergone a specific gene expansion (*n* = 45 in MJ and *n* = 35 in MP), which is also the largest expansion event in the analysis. Mammal-wide phylogenic analysis indicates that the expanded proteins constitute a de novo subfamily distinct with others, and only three subfamilies including *KRTAP7* (*n* = 1), *KRTAP8* (*n* = 1), and pangolin-specific HGT-KRTAPs are identified in contrast with seven subfamilies universal in other mammals (fig. 4*A*and*B*). The pangolin-specific proteins can be further classified into two types forming a large tandem array locus in the genome: 1) HGT-KRTAPs-t1s, the predominant type, adjacent to high sulfur KRTAP13s, constituting one side boundary of pangolin HGT-KRTAPs and 2) HGT-KRTAPs-t2s, adjacent to *KRTAP7* and *KRTAP8* in the genome, constituting the other side boundary (fig. 4*C*). HGT-KRTAPs-t2 is also featured by the unique 5′ terminal sequences, started with “MGY,” unique among all mammal HGT-KRTAP proteins. Interestingly, this gene cluster is firstly identified in the pangolin genome, which only limited number and fragmented HGT-KRTAPs sequence are identified in the previous assembly version.

To explore the origin of tandem array locus, we compared the mean percent amino acid identity among neighboring genes within the HGT-KRTAPs array of pangolins and human. The mean amino acid identities of neighboring genes of pangolins are significantly higher than human (one-way ANOVA, *P* < 0.0001), while it is close between two pangolins (*P* = 0.8707) (fig. 4*D*). Strikingly, the mean amino acid identity of pangolins is above 0.98 in contrast with 0.51 in human. Low identity of neighboring HGT-KRTAPs in mammals is expected due to anciently originated subfamilies and positive selection resulting diversification (Khan et al. 2014). High identity of HGT-KRTAPs array in pangolins could be explained by large number of specific HGT-KRTAPs and identical paralogs. For example, MJ_t1_a and MP_t1_a represent nonrepetitive gene clade respectively in Malayan pangolin and Chinese pangolin constituted by 32 and 27 identical paralogs (fig. 4*E*, supplementary table S15, Supplementary Material online). We calculate pair-wise synonymous substitution (Ks) within each t1_a clades indicating close to 0 of mean Ks values (Ks_MJ_ = 0.006, Ks_MP_ = 0.000) within both pangolins, and 0.03 between two species indicating the divergence of 7.5 million years conforming to the speciation time (Choo et al. 2016) (fig. 4*F*). The origin of the highly specific and homogenized HGT-KRTAPs in pangolins can be explained by two alternative scenarios. Firstly, they have been experienced independent and very recent duplications on both pangolins under the assumption of a molecular clock assuming a linear increase of the number of substitutions over time. However, this scenario could not explain the complete deletion of other HGT-KRTAP sub-families in the short time in which even pseudogenes could not be identified. Secondly, they have ancient origin shared among all extant pangolins tracing back to the first formation of scale, and persistent nonallelic gene conversion and selection jointly homogenized all HGT-KRTAP paralogs. Nonallelic gene conversion mediates the transfer of genetic information between nonallelic gene copies that contains the DNA double-strand breaks (Chen et al. 2007; Fawcett and Innan 2011). Large-scale gene conversion is rarely identified in mammals, only well studied in ribosomal RNA (rRNA) genes tandem array locus and male-specific region (MSY) on the Y chromosome (Chen et al. 2007). Pangolin HGT-KRTAPs tandem array has multiple characters confirming the consequences of gene conversion, including highly homogenized neighboring genes, tail-to-head orientations and elevated GC content in codon (fig. 4*G*and*H*). However, we could not completely exclude recent independent duplication. The origin of pangolin HGT-KRTAPs could be well tested by improving the assemblies of African pangolins and comparing among distant pangolins in the further study.

Pangolin-specific HGT-KRTAPs have overall shorter mean p-distance with human *KRTAP19* subfamily (supplementary figs. S9–S11, Supplementary Material online), while no clear orthologous relationships could be obtained to determine specific protein neither on collinearity nor phylogenetic tree, making it hard to characterize sequence evolution by codon models. We speculate that the selection may act by elevating the particular residues proportion of pangolin HGT-KRTAPs, where the high proportion of total glycine and tyrosine residues are identified (fig. 4*I*). Glycine has the smallest sidechain of any amino acid and has near complete freedom of rotation about the mainchain single bonds. On the other hand, tyrosine residues are large amino acids interacting strongly with each other through the formation of hydrogen bonds or “ring stacking” involving attractions between the aromatic rings, supposed to strengthen material (Fraser and Parry 2018). The both extremely high number of genes and high proportion of glycine and tyrosine reveal the strong connection between the scale formation and the evolution.

### Differential Analysis Verifying the Genes Relating Dorsal Scale Development of Pangolin

**Fig. 5.**
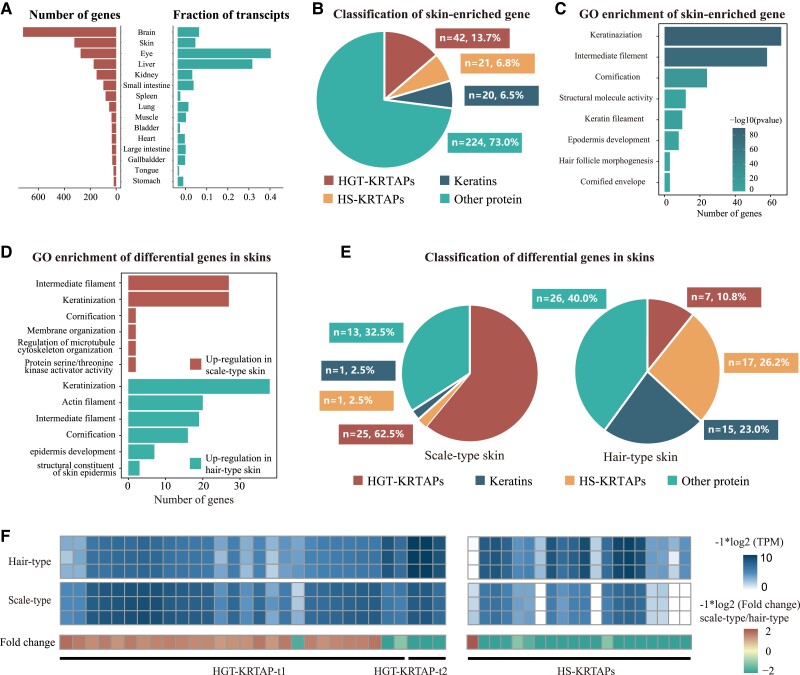
Differential analysis illustrating the scale formation mechanism. (*A*) The number of tissue-enriched genes in the 15 organ systems, and the fraction of all transcripts (TPM) encoded by these elevated genes for each of these organ systems. (*B*) Classification of skin-enriched genes, including the number and proportion of HGT-KRTAPs, HS-KRTAPs, KRTs, and other genes. (*C*) GO enrichment of skin-enriched genes. (*D*) GO enrichment of up-regulated genes in scale-type skin (up) and up-regulated genes in hair-type skin (down). (*E*) Classification of up-regulated genes in scale-type and hair-type skin. (*F*) Heat map showing the expression level and fold change between hair-type skin and scale-type skin.

To comprehensively validate proteins with key role in scale formation, we focus on those genes with skin-enriched expression manner and combine the differential expression analysis on both skins and skin appendages. In pangolin, the number of tissue-enriched genes in brain (*n* = 705) and skin (*n* = 303) are predominant, corresponding to 3.3% and 1.42% of all genes, respectively (fig. 5*A*). It is expected that brain have the largest number of enriched genes that is also consistent in human tissues (Uhlén et al. 2015). The abundant genes with skin-enriched expression manner are likely specific in pangolin partly owing to the large number of HGT-KRTAPs (fig. 5*B*). The functional GO analysis for skin-enriched genes is consistent with the function of the formation of keratin and epidermis, such as “keratinization” (*P* = 5.1 − 107), “cornification” (*P* = 2.7 − 20), and “structural constituent of skin epidermis” (*P* = 8.8 − 08) (fig. 5*C*, supplementary table S16, Supplementary Material online). To obtain genes concerning scale or hair development, we further compare the gene expression between dorsal (scale-type) and abdominal (hair-type) skin. A total of 104 differential expression genes are identified (fold change >2 and adjusted *P*-value <0.05 and skin enriched), of which 40 genes are up-regulated in scale-type skin, and the remaining 65 genes were up-regulated in hair-type skin (fig. 5*D*, supplementary tables S17–S20, Supplementary Material online). It can be observed that both gene sets are significantly enriched in the pathway concerning keratinization, where HGT-KRTAPs and high cysteine HS-KRTAPs account for more than half of them yet asymmetric distribution between two types of skin (fig. 5*E*). Indeed, all pangolin specific HGT-KRTAPs have skin enriched expression manner, and most of them have significantly higher expression on scale-type skin. HGT-KRTAPs-t1s are dominant in scale-type skin in contrast to HGT-KRTAPs-t2s on hair-type skin (fig. 5*F*, supplementary fig. S12, Supplementary Material online). Unlike HGT-KRTAPs dominantly expressing on scale-type skin, 15 HS-KRTAPs derived from 8 subfamilies with significantly higher expression level on hair-type skin. KRTAPs are supposed to participate in the formation and diversification of skin appendages, such as nail, and hairs, and only express in the hair follicle, hair cortex, and hair cuticle (Rogers et al. 2006). The divergent expression pattern of KRTAPs in pangolin is mostly derived from the function specialized cells in hair follicle contributing the formation of hair or scale rather than different structure of the epidermal and hypodermis.

To quantify the protein abundance in skin appendages, we perform label-free quantification on pangolin dorsal scale and ventral hair by high-resolution mass spectrometry (MS)/MS acquisition. There are 13 proteins significantly enriched in scale and 26 proteins enriched in hair (fold change >2 and *q*-valueFDR <0.05 and skin enriched) (supplementary table S21, Supplementary Material online). Consisting with the RNA-seq from skins, KRTAPs are still the primary differentially abundant proteins, including six up-regulated proteins in scale: HGT-KRTAPs (t1 type) and HS-KRTAPs (*KRTAP1-3*, *KRTAP3-3*, *KRTAP13-1*, and *KRTAP27-1*), as well as five up-regulated proteins in hair: HGT-KRTAPs (t2 type) and HS-KRTAPs (*KRTAP13-2*, *KRTAP9-1*, *KRTAP26-1*, and *KRTAP24-1*). Besides, keratins constitute another important part of differentially abundant proteins, including type II (neutral to basic) keratins, *KRT85* and *KRT84*, and type I (acidic) keratins, *KRT31* enriched in scale. These KRTs are hair keratins, expressing only in highly keratinized tissues such as the hair shaft and nails in mammals. Interesting, *KRT85* can form keratin intermediate filaments with *KRT31* through heterodimerization in the lower portion of the human hair cortex. The formation of a hard and compact hair fiber can be achieved by multiple cross-linkings of keratin intermediate filaments with multiple KRTAP proteins where the significantly abundant KRTAPs in scale are the most convincing factor contributing the hardness character.

In general, pangolin-specific HGT-KRTAPs have the strongest evolutionary feature directing their role in the hardness character of pangolin scale. HS-KRTAPs also likely contribute the character by forming so-called “β-keratin.” Previous XRD studies reported that the pangolin scale contains both α- and β-keratins, a feature of Sauropsida appendages such as scale and feather, and pangolin β-keratins are supposed to the key component for hardness character(Meyer et al. 2013; Wang et al. 2016). However, we do not identify any pangolin sequences or proteins homolog to β-keratins in reptiles or birds by mapping the pangolin protein sequences to those in Pfam keratin family (PF02422). We also do not identify one type of proteins in pangolin scale or skin with both high level of expression and unidentified protein characters in database. Therefore, there have strong evidence indicating that the β-keratin proteins in pangolin scale are derived from shared mammal proteins rather than particular de novo proteins. To identify pangolin β-keratins, we predict the secondary structure of those genes with differential expression in scale using Psipred (McGuffin et al. 2000). Low level of β-sheet region is also found in the KRTs similar with other mammals. On the other hand, some of HS-KRTAPs have higher proportion of β-sheet regions, up to 20% in *KRTAP3-3*, highly indicating the origin of pangolin β-keratin. However, we identify consistent high level of β-sheet region in human *KRTAP3-3* proteins that have specific expression in human hair cortex (supplementary table S22, Supplementary Material online). Although proteins with β-sheet regions can be found in both pangolin scale and human hair, the β-keratin curves of XRD pattern are only found in pangolin scale. It raises an interesting hypotheses about the nature of the pangolin scale β-keratin that is formed by the over-expression of homolog HS-KRTAPs resulting qualitative change rather than pangolin specific protein structure. It could be tested by directing quantification between pangolin scale and hair in other mammals in further researches.

## Discussion

We present the most integrate and accurate pangolin genomes for both the Chinese pangolin and Malayan pangolin, and our RNA sequencings on Malayan pangolin are the most comprehensively analyzed to date. By combining the results from multi-omics, evolution, and fundamental proteins feature analysis, we provide credible molecular evidence on the genes acting on the myrmecophagous diet and defensive armor formation.

The first assemblies of pangolins (Choo et al. 2016) was submitted in 2016 that identified many pseudogenized genes concerning epithelial cell development and immunity by comparing the gene structure and selection. However, short reads based assemblies limited the effect of identifying complex gene clusters, such as EDC and KRTAPs. The defect was remained even in the assembly applying 10× Genomics scaffolding technology (Hu, Hao, et al. 2020). The genes numbers in both EDC and HGT-KRTAPs cluster are significantly improved compared with NCBI published assemblies. We identified 16 and 24 Malayan pangolin EDC genes in published and current version respectively, in which *LCEs* are completely missing. In HGT-KRTAPs cluster, Malayan pangolin published version only identified one pangolin specific HGT-KRTAP, indicating severe collapsed assembly in the region. It is worth to point that we also observed unanchored contigs in chromosomes level assemblies, located in EDC and KRTAP cluster, probably due to scaffolding process using Hi-C data. To obtain more creditable results, we use contig assemblies for most analysis to avoid potential errors on comparison among species.

We identified a pangolin specific rearranged EDC organization compared with other mammal genomes. In fact, conserved gene structures and organizations can be observed even between mammal and amniotes, and the changes on sequences and expression patterns of EDC-related genes contribute to the evolution of turtle shell and bird feather (Holthaus et al. 2016). We determined the skin-stomach coexpression pattern on Malayan pangolin EDC genes, especially SEDCs, which are skin enriched in both human and cattle. There is clear evidence on the decay of pangolin CNEs acting on the expression reversions. In addition, downstream cornification genes participating cornified envelopes and intercellular lipids are also identified participating the cornification in stomach, thus, indicating an integrate cornification pathway. Since the discovery of pangolin myrmecophagous diet, the roles of microbiota and digestive enzymes in gut contents have been especially highlighted in multiple researches with the aim of interpreting the hologenomic mechanisms (Zhang, Xu, et al. 2019; Liu et al. 2021; Cheng et al. 2022). Our study firstly illustrate the stomach cornified structure to address the specific pangolin evolution supported by genetic and transcriptomic variation for the adaption.

Benefited by comprehensive gene annotation, gene family analysis allows us to firstly address particular evolutionary process of HGT-KRTAPs, the expansion events absent both in HS-KRTAPs and KRTs. We also make progress on the differentiation analysis on the scale and hair type tissues by adding tissues enriched analysis. On microscopic observation (Liumsiricharoen 2008), the pangolin dorsal skin and scale are tightly connected with muscle, yet absent on the abdominal skin and hair, resulting mixed component of scale type skin samples on previous RNA-seq analysis (Li et al. 2020). The identification of skin enriched genes overcome the limitation, and further facilitate the identification of hair follicle specific expression proteins that are absent in the epithelial cell of other organs. All HGT-KRTAPs have skin enriched expression manner and the vast majority of them are significantly up-regulated on scale-type skin also supported by the proteome data between scale and hair. Meanwhile, the extremely high portion of both glycine and tyrosine in pangolin scale indicates the most convincing molecular origin of HGT-KRTAPs excluding low level of these residues among all types of KRTs (Tong et al. 1995). We also underline the mass component of *KRT85*, *KRT84*, and *KRT31* in scales, rather than previous identified *KRT36* by lacking skin enriched expression pattern. However, we have not additional evidence to reveal their role in scale hardness. In conclusion, we infer HGT-KRTAPs as the most important proteins in the pangolin scale formation, the only proteins satisfying all the conditions listed above. Besides, we observed high level of homogeneity and tandem array organization of the HGT-KRTAPs, likely formed by ancient large range gene conversion or recent independent expansion. However, it is still unknown that the nature of pangolin scale hardness is due to the improved gene structure of HGT-KRTAPs having more efficient binding effect with intermediate filaments or due to elevated content serving as structural proteins. Uncovering the inherent pangolin scale formation principles could inspire the related biomimetics and further solve the engineering problems (Li et al. 2019; Wang et al. 2021). Our discovery of the evolution for pangolin scale formation may have revealed plausible molecular mechanism that may explain how the highly specialized morphological characters are derived by the environment.

## Materials and Methods

### Sample Collection and De Novo Sequencing

All animal work was approved by the Guangxi Academy of Forestry Sciences. For the reference genome sequencing, both pangolins were derived from the artificial pangolin farms of the Guangxi Academy of Forestry Sciences. When we extracted the samples, they have been dead due to natural causes. DNAs were extracted using a QIAGEN DNeasy (DNA) kit (Qiagen, Hilden, Germany). Three de novo genome sequencing methods were performed on the Chinese pangolin and Malayan pangolin: 1) 263 Gb (∼104× depth) and 264 Gb (∼108× depth) Oxford Nanopore long reads, with an average read length of 15.8 kb (PromethION platform); 2) 253 Gb (∼98× depth) and 230 Gb (∼94× depth) Illumina HiSeq PE150 pair-end sequencing to correct errors (Illumina, San Diego, CA, USA); and 3) 287 Gb (∼113× depth) and 280 Gb (∼115× depth) chromosome conformation capture sequencing (Hi-C) data (sequenced by Illumina platform).

### De Novo Assembly and Assessment of the Genome Quality

Assembly was performed using Wtdbg2 (Ruan and Li 2020) using fuzzy Bruijn graph based assembly algorithms. It uses raw reads without error correction and then builds the consensus from intermediate assembly output. NextPolish (Hu, Fan, et al. 2020) was used to improve the local base accuracy of the contigs via analysis of the read alignment information based on paired-end bam and long-reads bam files. The procedure is repeated thrice. As a result, the initial assembly resulted had an N50 size of 15.81 and 13.97 Mb for Malayan and Chinese pangolins respectively. ALLHiC (Zhang, Zhang et al. 2019) was capable of building chromosomal-scale scaffolds for the initial genome using Hi-C paired-end reads containing putative restriction enzyme site information. The whole genome assembly (contig version) has been deposited in the NCBI under Bioproject PRJNA813723 and PRJNA822507.

Three methods were used to evaluate the quality of the genomes. First, we used QUality ASsessment Tool (QUAST) (Mikheenko et al. 2018) to align the Illumina and Nanopore raw reads to the two pangolin reference genomes to estimate the coverage and mapping rate. Second, all the Illumina paired-end reads were mapped to the final genome using BWA (Li and Durbin 2010), and single nucleotide polymorphisms were called using SAMtools and BCFtools (Li et al. 2009). The predicted error rate was calculated by the homozygous substitutions divided by length of the whole genome, which included the discrepancy between assembly and sequencing data. Thirdly, we assessed the completeness of the genome assemblies and annotated the genes using BUSCO (Waterhouse et al. 2018).

### RNA-seq Collection and Genome Annotation

Gene expression data were produced to improve the gene annotation and followed analysis. Sixty-five tissues samples from a male Malayan pangolin were collected. RNA was prepared using RNeasy isolation kit (Qiagen). RNA integrity was assessed using an Agilent Technologies 2100 Bioanalyzer, and samples with an RNA Integrity Number value >8.5 were used to prepare libraries with an mRNA-seq sample preparation kit (Illumina Inc.) according to the manufacturer's protocol. The quality of the data was explored by mapping transcript sequences to constructed Malayan pangolin genome sequence using HISAT2 (Kim et al. 2019). The raw reads were processed and analyzed using transcriptome assembly pipeline based on the StringTie software (Pertea et al. 2015).

Three gene prediction methods, based on de novo prediction, homologous genes, and transcriptomes, were integrated to annotate protein-coding genes. Transcription assemblies are initially performed by StringTie illustrated above. PASA (v2.4) (Talavera and Castresana 2007) was another tool used to assemble RNA-seq reads and further generated gene models to train de novo programs. Two de novo programs, including Augustus (v3.0.2) (Stanke et al. 2004) and SNAP (v2006-07-28) (Krogh et al. 2001), were used to predict genes in the repeat-masked genome sequences. For homology-based prediction, protein sequences from relative species, including pangolins, dog, human, cattle, and horse, downloaded in NCBI were uploaded on exonerate (Slater and Birney 2005) to identify gene structures. All predicted genes from the three approaches were combined using MAKER (Holt and Yandell 2011) and EVM (Haas et al. 2008) to generate high-confidence gene sets. To obtain gene function annotations, InterProScan (v5.45) (Jones et al. 2014) was used to identify annotated genes features, including protein families, domains, functional sites, and GO terms from the InterPro database. SwissProt and TrEMBL protein databases were also searched using BLASTp (Altschul et al. 1997). The best BLASTp hits were used to assign homology-based gene functions. KEGG web was used to search the KEGG ORTHOLOGY (KO) database (Kanehisa and Goto 2000). The subsequent enrichment analysis was performed using clusterProfiler using total annotated genes as the background with the “enricher” function (Yu et al. 2012).

The alignment between pangolin and human genomes is constructed by Minimap2 (Li 2018). The gene loci of KRTs, HGT-KRTAPs, and EDC clusters are firstly determined by the genome alignment. The previous protein annotation is firstly filtered based on the gene structure and expression on whole tissues. Transcription assembly from skin samples is extracted and candidate coding regions within the assembled transcripts were predicted using the TransDecoder program (http://transdecoder.sourceforge.net/). The best BLASTp hits of newly predicted genes were used to determine their function. The related genes were extracted and performed homolog based prediction to identify paralogues using genBlastG (She et al. 2011). The procedures will perform multiple times.

### RNA-Based Analysis

To obtain quantification scores for all human genes and transcripts across all 65 samples, read counts were calculated by featureCounts (Liao et al. 2014) and TPM (transcript per million) values were calculated using custom scripts. Each of annotated genes were classified into 1 of 3 categories based on the TPM levels in 12 tissues or organs system: 1) “Not detected”: TPM < 1 in all tissues; 2) tissue enriched: at least a 5-fold higher TPM level in one tissue compared with all other tissues; and 3) group enriched: 5-fold higher average TPM value in a group of tissues (*Z*-score ≥1) compared with all other tissues (*Z*-score <1). The differential expression analysis between scale-type skin (dorsal) and hair-type skin (abdominal) was performed using the DEGseq R package (Wang et al. 2010). Differential genes were further filtered by *P*-value, average fold change and skin enriched expression manner.

### Gene Family Analysis

We chose the longest transcript in the downloaded annotation dataset to represent each gene, and removed genes with open reading frames shorter than 150 bp. Gene family clustering was then performed using OrthoFinder (v 2.3.12) (Emms and Kelly 2019), based on the predicted gene set for nine genomes, including Chinese pangolin, Malayan pangolin, dog (NCBI: GCF_014441545.1), cattle (NCBI: GCF_002263795.1), horse (NCBI: GCF_002863925.1), mouse (NCBI: GCF_000001635.27), dasypus (NCBI: GCF_000208655.1), elephant (NCBI: GCF_000001905.1), and human (NCBI: GCF_000001405.39). This analysis yielded 28,318 gene families. To identify gene families that had undergone expansion or contraction, we applied the CAFE (v5.0.0) program (Han et al. 2013), which inferred the rate and direction of changes in gene family size over a given phylogeny.

OrthoFinder indicates that pangolin-specific HGT-KRTAPs formed an individual family due to low similarity with other proteins. To identify the homologs of pangolin HGT-KRTAPs, we combined the results of synteny and the identity human HGT-KRTAPs. Distance matrix was constructed using MEGA (Tamura et al. 2007). Phylogenetic trees is constructed using RAxML and IQTREE (Stamatakis 2014; Minh et al. 2020). Pair-wise Ks value were calculated by codon-based codeml PAML with “runmodel = −2” (Yang 2007). Divergent time is estimated by the formula: *T* = Ks/2mu, mu = 2 × 10^−9^ substitution per year.

### Label-Free Quantitative Protein Analysis

The resulting spectra from pangolin scale and hair samples were searched against our annotation database using the search engine Proteome Discoverer 2.2 (PD 2.2, Thermo). A maximum of two missed cleavage sites was allowed. Identified proteins contained at least one unique peptide with FDR no more than 1.0%. Proteins containing similar peptides that could not be distinguished by MS/MS analysis were identified as the same protein group. Precursor ion was quantified by the label-free method based on intensity and was used for label-free quantification. Proteins whose quantitation significantly differed between scale and hair (*P* ≤ 0.05, average fold change ≥2, and skin-enriched expression manner) were defined as differentially expressed proteins.

## Supplementary Material

msac262_Supplementary_Data

## Data Availability

The data supporting the findings of this work are available within the paper and its Supplementary Information files. The genomes generated in this study have been deposited in the NCBI under accession code PRJNA813723 and PRJNA822507.
