# Supplementary material for: High-Quality Genomes of Pangolins: Insights into the Molecular Basis of Scale Formation and Adaption to Myrmecophagous Diet
Source: Mol Biol Evol. 2022 Dec 31;40(1):msac262. doi: 10.1093/molbev/msac262 (PMC9848057; doi:10.1093/molbev/msac262)
Supplement: msac262_Supplementary_Data [file msac262_supplementary_data.zip › Suppl Fig.pdf]

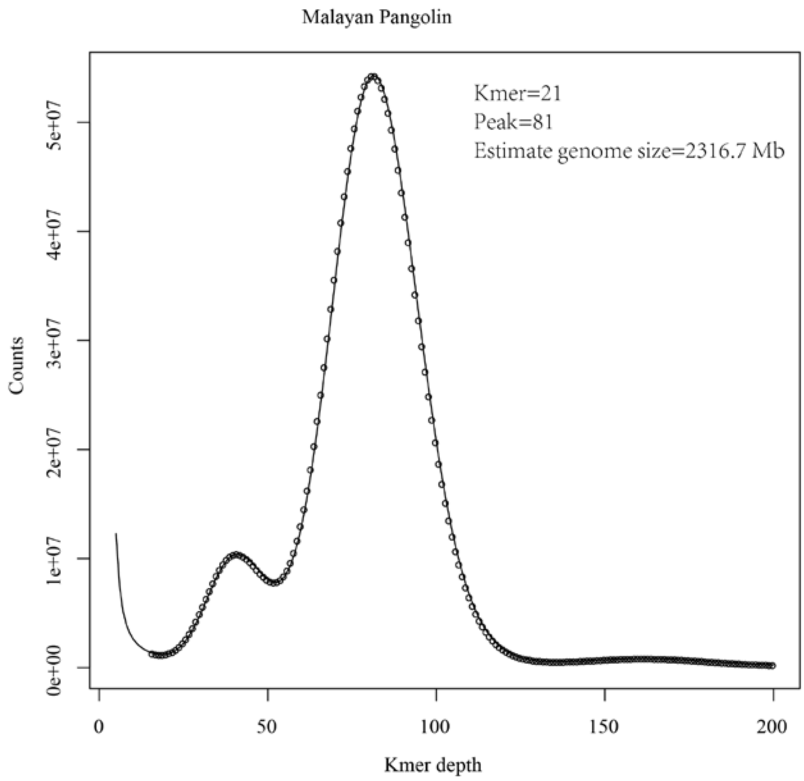

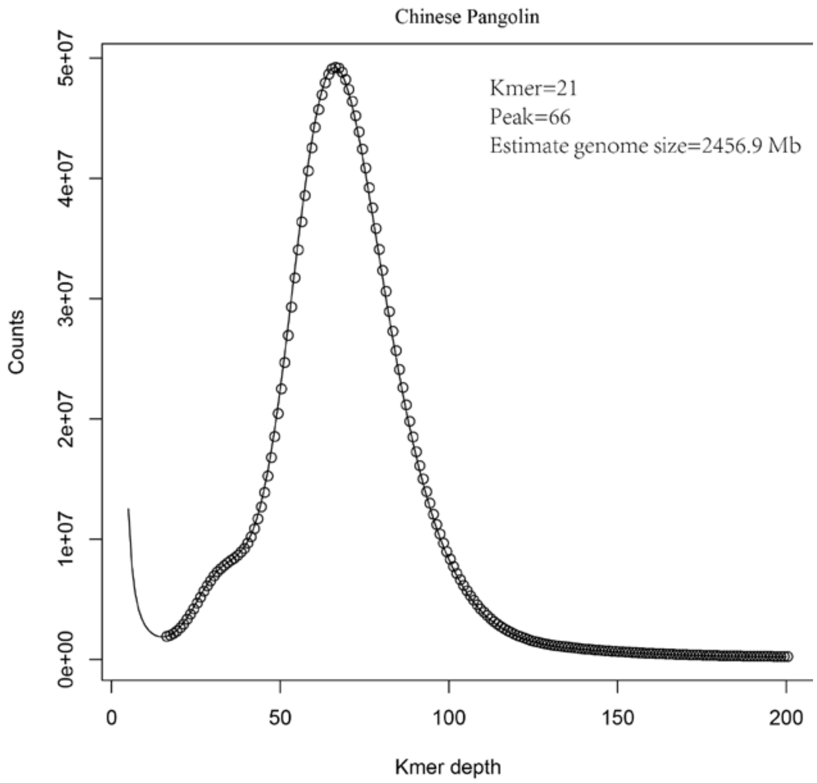

Fig. S1 Genome size estimation for Malayan pangolin and Chinese pangolin using Illumina reads by Kmer 21.

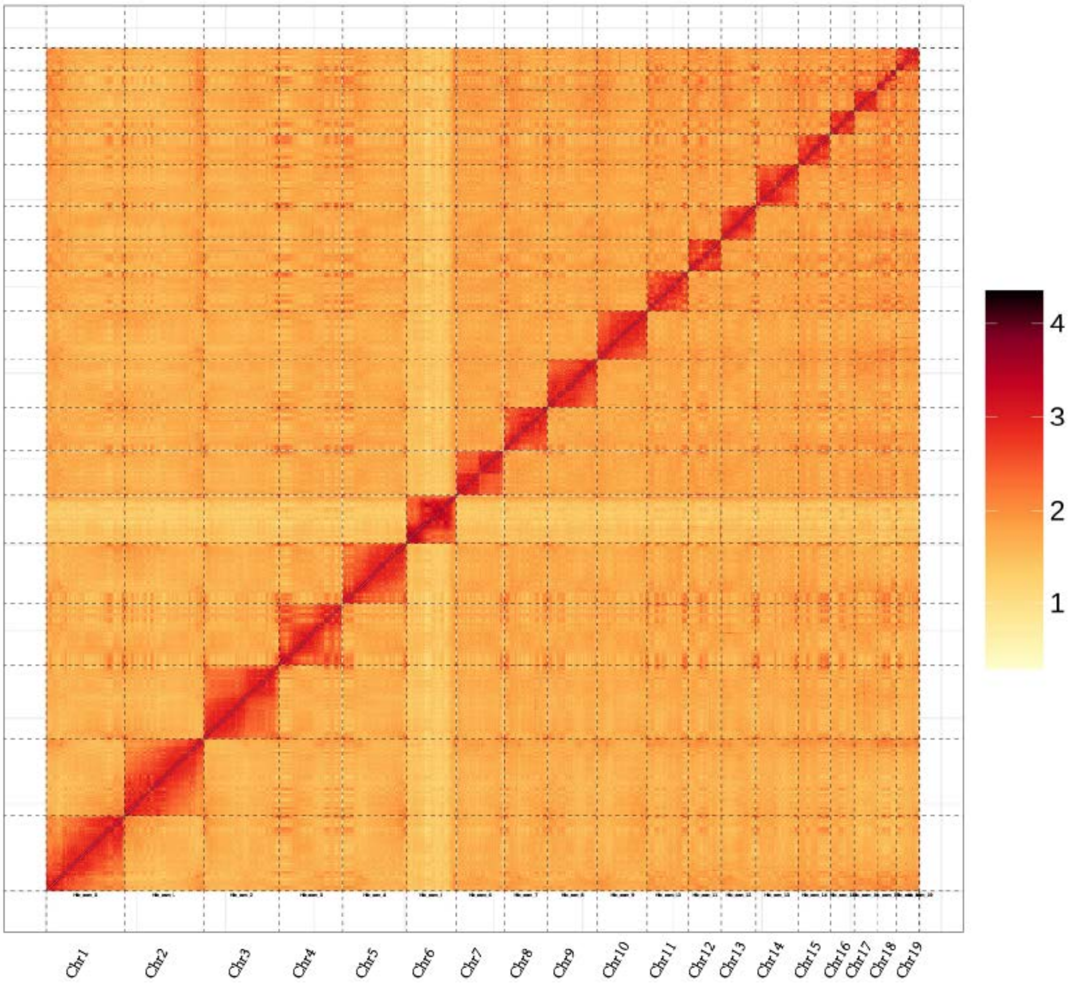

Fig. S2 Genome-wide all-by-all Hi-C interaction in Chinese pangolin

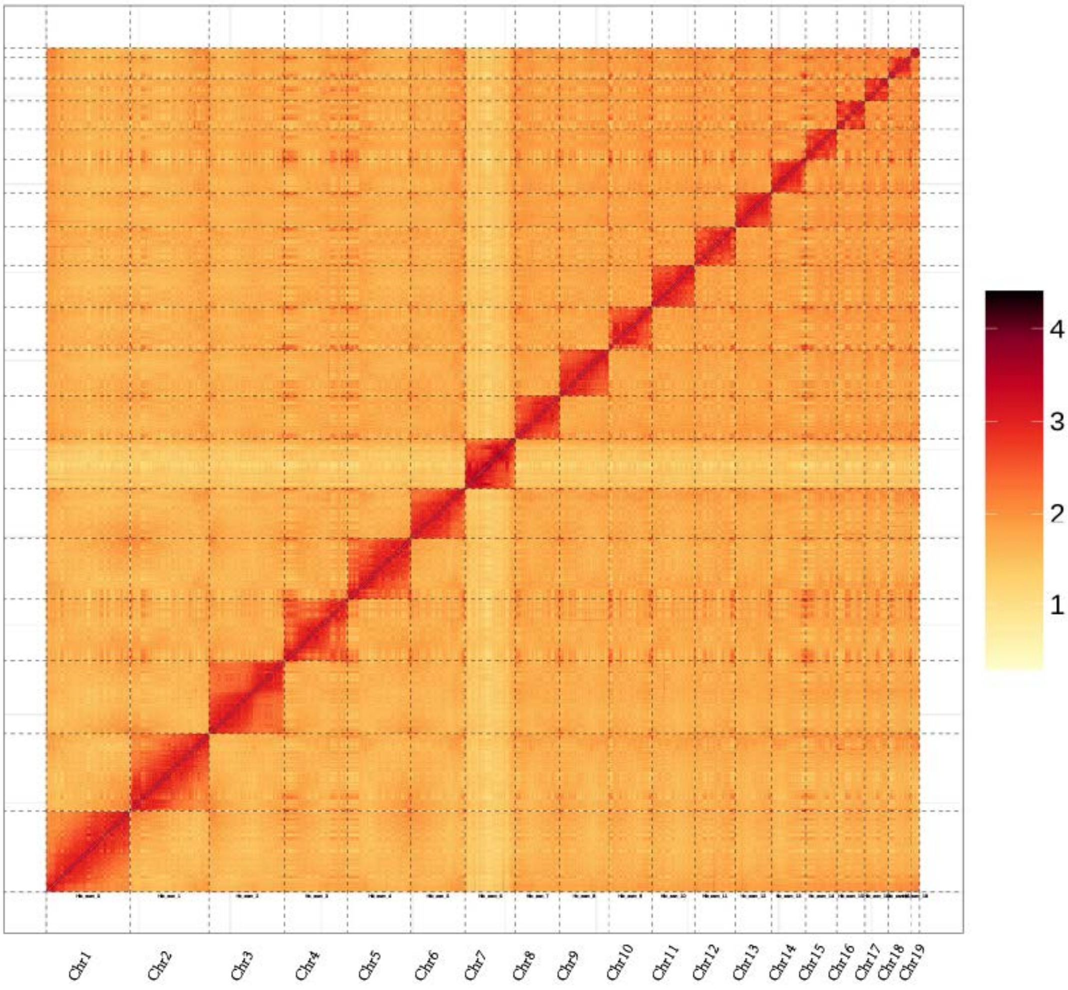

Fig. S3 Genome-wide all-by-all Hi-C interaction in Malayan pangolin

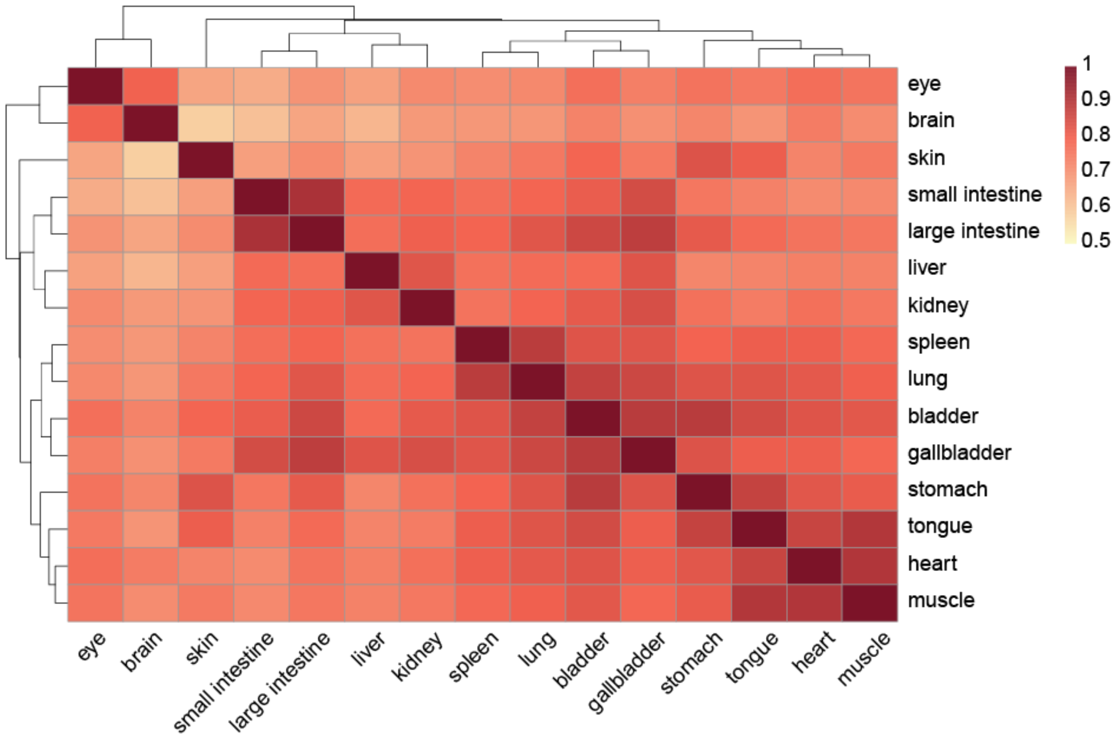

Fig. S4 Heat map showing the pairwise correlation between 15 main tissues based on transcript expression levels of all genes in Malayan pangolin. The average TPM values for each gene and tissue are used in the analysis.

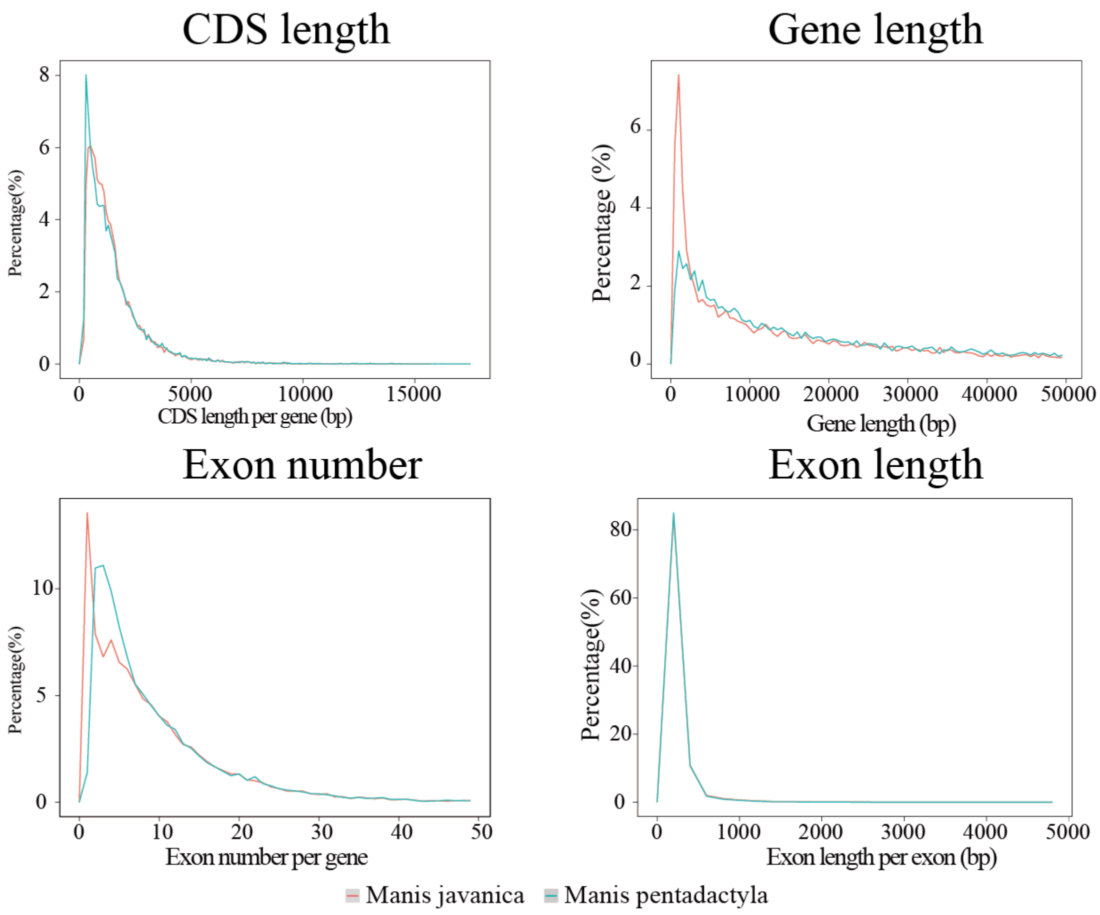

Fig. S5 Comparison of gene structures between Chinese pangolin and Malayan pangolin.

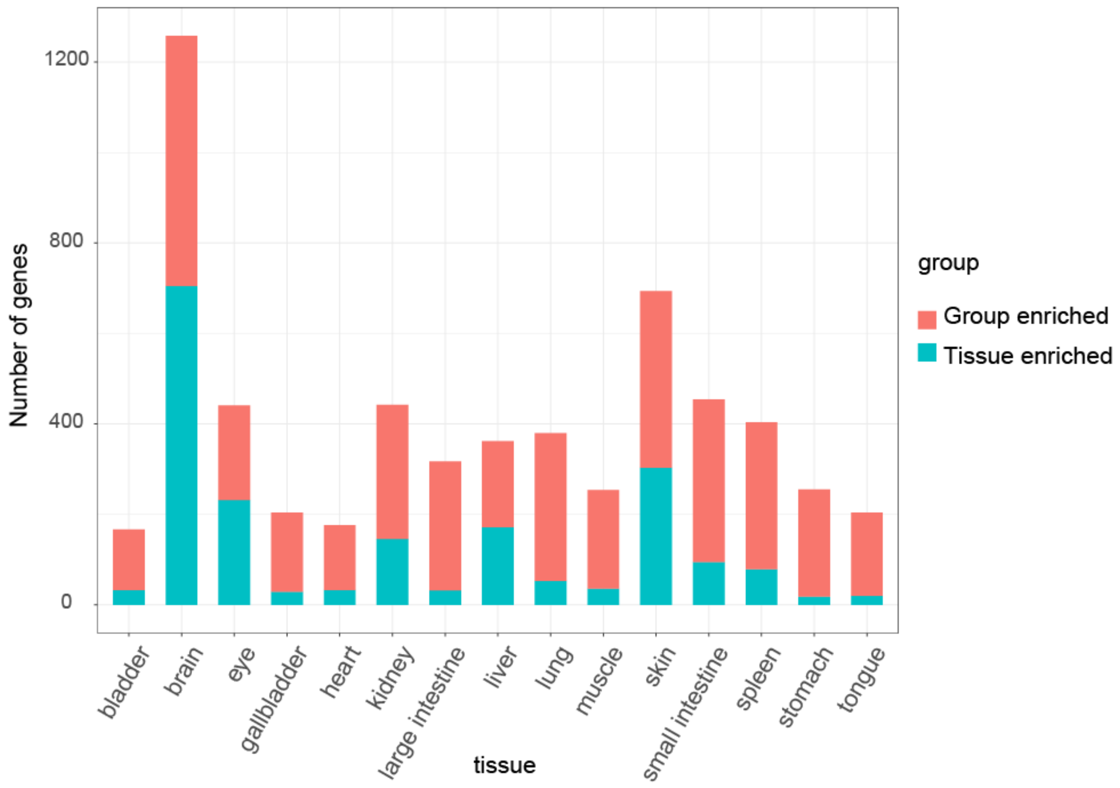

Fig. S6 Number of genes with tissues specification

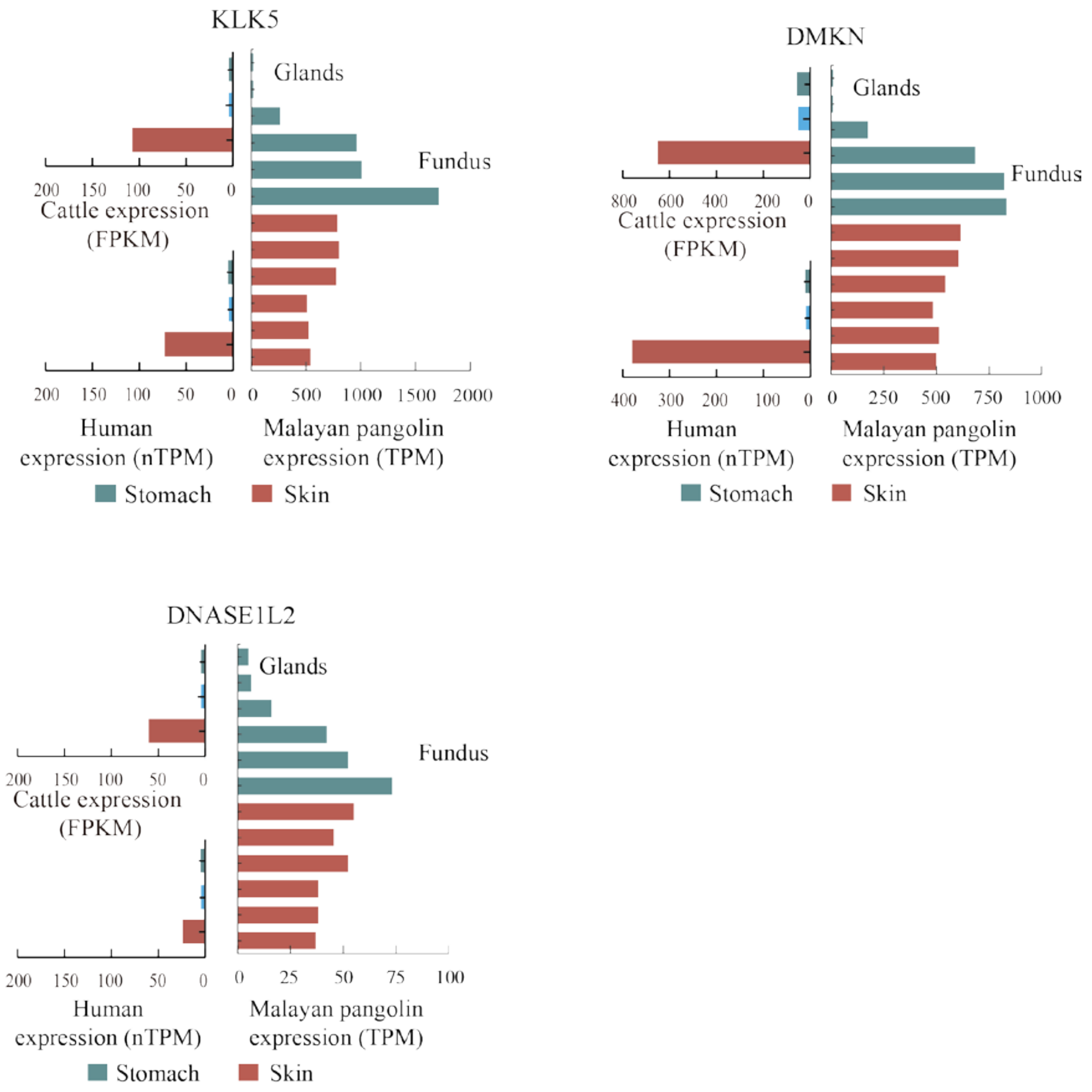

Fig. S7 Expression (TPM or nTPM) of KLK5, DMKN, and DNASE1L2 in the stomach and skin.

Amino acid	Pangolin scale (%)	Keratin in hair (%)	Keratin in feather (%)
**Glycine**	**10.6-13.7**	**5.2-6.5**	**7.2**
Alanine	2.3-3.0	3.4-4.4	5.4
Valine	3.2-4.0	5.0-5.9	8.8
Leucine	7.8-7.9	7.6-8.1	8
Isoleucine	2.0-2.3	3.1-4.5	6
Serine	5.0-5.5	7.2-9.5	14
Threonine	3.2-3.4	6.6-6.7	4.8
**Tyrosine**	**17.2-20.0**	**4.0-6.4**	**2.2**
Phenylalanine	3.0-3.2	3.4-4.0	5.3
Cysteine	1.9	-	-
Cystine	-	11.4-14.1	8.2
Methionine	0.4-0.6	0.5-0.7	0.5
Tryptophan	0.4 — 0.5	1.8-2.1	0.7
Arginine	8.0-8.6	9.2-10.0	7.5
Histidine	0.7-1.2	2.8-3.3	0.7
Lysine	2.5-2.6	0.7-1.1	1.7
Aspartoyl	7.1 — 7.9	6.4-7.3	7.5
Glutamic acid	10.4-10.9	13.1-16.0	9.7
Proline	8.6-9.7	5.8-8.1	10

Fig. S8 Amino acids contents estimated by Tong etc. (Tong, J., Ren, L. Q. & Chen, B. C. Chemical constitution and abrasive wear behaviour of pangolin scales. Journal of Materials Science Letters 14, 1468-1470, doi:10.1007/BF00462216 (1995).)

		1	2	3	4	5	6	7	8
Pangolin	1. HGT-KRTAPs								
Human	2. KRTAP19	0.274							
	3. KRTAP20	0.329	0.319						
	4. KRTAP22	0.542	0.458	0.472					
	5. KRTAP6	0.500	0.458	0.454	0.542				
	6. KRTAP21	0.599	0.595	0.583	0.672	0.525			
	7. KRTAP8	0.800	0.829	0.857	0.900	0.827	0.674		
	8. KRTAP7	0.625	0.685	0.676	0.750	0.569	0.773	0.889	

Fig. S9 Mean p-distance matrix between pangolin specific HGT-KRTAPs and human sub-families.

	1	2	3	4	5	7	8	9	10
Malayan pangolin									
Chinese pangolin	0.082								
human KRTAP19-1	0.324	0.351							
human KRTAP19-2	0.509	0.497	0.365						
human KRTAP19-3	0.252	0.285	0.074	0.365					
human KRTAP19-4	0.445	0.441	0.452	0.529	0.388				
human KRTAP19-5	0.342	0.341	0.208	0.365	0.153				
human KRTAP19-6	0.353	0.365	0.190	0.447	0.172	0.259			
human KRTAP19-7	0.302	0.331	0.222	0.349	0.159	0.222	0.204		
human KRTAP19-8	0.330	0.343	0.222	0.372	0.159	0.222	0.286	0.222	

Fig. S10 P-distance matrix between pangolin specific HGT-KRTAPs and human KRTAP19

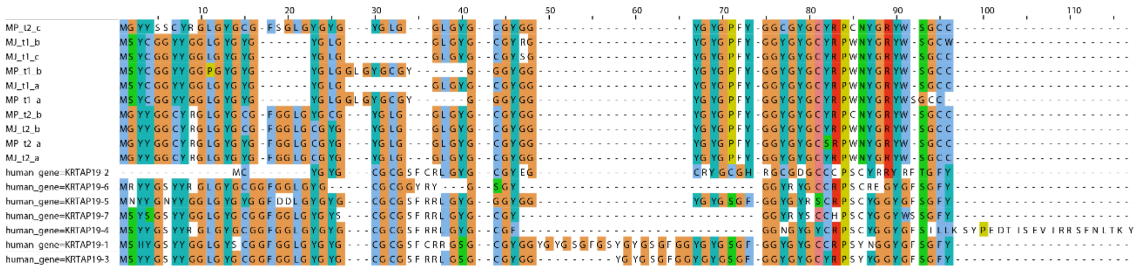

Fig. S11 Multiple sequence alignment among pangolin specific HGT-KRTAP and human KRTAP19.

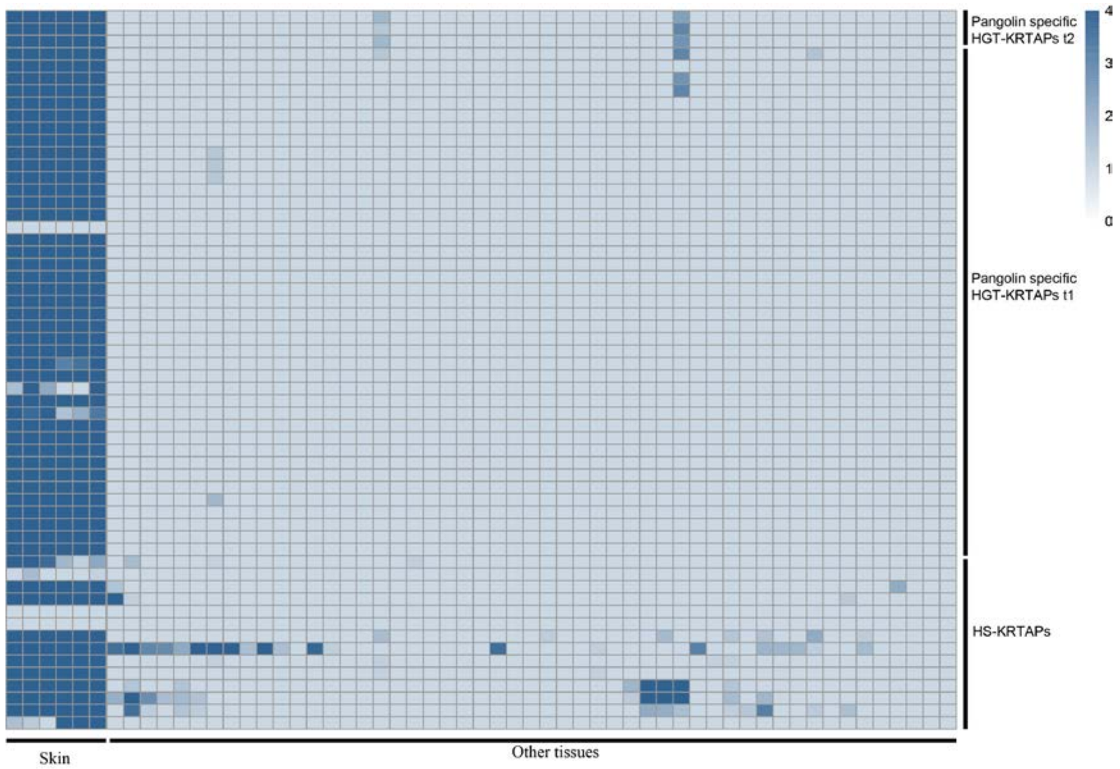

Fig. S12 Expression (TPM, log10-transformed) of KRTAPs across tissues.
